# Supplementary material for: MetaRibo-Seq measures translation in microbiomes
Source: Nat Commun. 2020 Jun 29;11:3268. doi: 10.1038/s41467-020-17081-z (PMC7324362; doi:10.1038/s41467-020-17081-z)
Supplement: Supplementary file 10 — Supplementary Data 7 [file 41467_2020_17081_MOESM10_ESM.zip › File2/Confidence_VeryHigh_Taxonomy/3764_out.krona.html]

Javascript must be enabled to view this page.

members
magnitude
magnitudeUnassigned
count
unassigned
taxon
rank

3764\_out

7

2
superkingdom
7

74201
phylum
2

class
203494
2

2
order
48461

2
1647988
family

genus
239934
2

species
239935
2

SRS077641\_contig\_number\_8987SRS147977\_contig\_number\_contig-100\_15017.15018

1239
phylum
5

5
186801
class

1
5

SRS014948\_contig\_number\_20091
order
186802

family
31979
3

3
genus
1485


SRS014313\_contig\_number\_4655SRS146764\_contig\_number\_31950SRS148721\_contig\_number\_48054
3
59620
species

family
541000
1

946234
genus
1

1

SRS104311\_contig\_number\_26959
species
1193534
